# A Smartphone-Based Self-management Intervention for Individuals With Bipolar Disorder (LiveWell): Protocol Development for an Expert System to Provide Adaptive User Feedback

**DOI:** 10.2196/32932

**Published:** 2021-12-24

**Authors:** Evan H Goulding, Cynthia A Dopke, Tania Michaels, Clair R Martin, Monika A Khiani, Christopher Garborg, Chris Karr, Mark Begale

**Affiliations:** 1 Department of Psychiatry and Behavioral Sciences Feinberg School of Medicine Northwestern University Chicago, IL United States; 2 Deparment of Pediatrics Loma Linda Children's Hospital Loma Linda, CA United States; 3 Rogers Behavioral Health Milwaukee, WI United States; 4 Audacious Software Chicago, IL United States; 5 Vibrent Health Fairfax, VA United States

**Keywords:** adaptive, personalized, self-management, smartphone, behavioral intervention technology, mHealth, bipolar disorder, depression, mania

## Abstract

**Background:**

Bipolar disorder is a severe mental illness that results in significant morbidity and mortality. While pharmacotherapy is the primary treatment, adjunctive psychotherapy can improve outcomes. However, access to therapy is limited. Smartphones and other technologies can increase access to therapeutic strategies that enhance self-management while simultaneously augmenting care by providing adaptive delivery of content to users as well as alerts to providers to facilitate clinical care communication. Unfortunately, while adaptive interventions are being developed and tested to improve care, information describing the components of adaptive interventions is often not published in sufficient detail to facilitate replication and improvement of these interventions.

**Objective:**

To contribute to and support the improvement and dissemination of technology-based mental health interventions, we provide a detailed description of the expert system for adaptively delivering content and facilitating clinical care communication for LiveWell, a smartphone-based self-management intervention for individuals with bipolar disorder.

**Methods:**

Information from empirically supported psychotherapies for bipolar disorder, health psychology behavior change theories, and chronic disease self-management models was combined with user-centered design data and psychiatrist feedback to guide the development of the expert system.

**Results:**

Decision points determining the timing of intervention option adaptation were selected to occur daily and weekly based on self-report data for medication adherence, sleep duration, routine, and wellness levels. These data were selected for use as the tailoring variables determining which intervention options to deliver when and to whom. Decision rules linking delivery of options and tailoring variable thresholds were developed based on existing literature regarding bipolar disorder clinical status and psychiatrist feedback. To address the need for treatment adaptation with varying clinical statuses, decision rules for a clinical status state machine were developed using self-reported wellness rating data. Clinical status from this state machine was incorporated into hierarchal decision tables that select content for delivery to users and alerts to providers. The majority of the adaptive content addresses sleep duration, medication adherence, managing signs and symptoms, building and utilizing support, and keeping a regular routine, as well as determinants underlying engagement in these target behaviors as follows: attitudes and perceptions, knowledge, support, evaluation, and planning. However, when problems with early warning signs, symptoms, and transitions to more acute clinical states are detected, the decision rules shift the adaptive content to focus on managing signs and symptoms, and engaging with psychiatric providers.

**Conclusions:**

Adaptive mental health technologies have the potential to enhance the self-management of mental health disorders. The need for individuals with bipolar disorder to engage in the management of multiple target behaviors and to address changes in clinical status highlights the importance of detailed reporting of adaptive intervention components to allow replication and improvement of adaptive mental health technologies for complex mental health problems.

## Introduction

Bipolar disorder is a severe mental illness characterized by episodes of mania, hypomania, depression, and mixed states [1,2]. Pharmacotherapy is the primary treatment for bipolar disorder, but even when pharmacological treatment is initially effective, high rates of episode recurrence, interepisode symptoms, and psychosocial impairment persist [3-7]. Combining psychotherapy with pharmacotherapy decreases episode recurrence and symptom burden while also improving quality of life [8-16], and treatment guidelines recommend providing access to psychotherapy for individuals with bipolar disorder [17-19]. Despite these recommendations and the demonstrated effectiveness of adjunctive psychotherapy, multiple barriers limit access to therapy, and only half of individuals with bipolar disorder receive any psychotherapy [20-24]. Enhancing access to tools and content derived from empirically supported psychotherapies thus has the potential to improve treatment for individuals with bipolar disorder.

Because smartphones are widely used and accepted for mental health assistance [25-29], smartphone-based mental health technologies (MHTs) provide a promising means for increasing access to tools and content derived from psychotherapy. In addition, individuals with bipolar disorder in sustained remission report using self-management strategies that overlap significantly with those delivered by empirically supported psychotherapies, and many people with bipolar disorder are interested in utilizing self-management strategies [30-33]. This suggests that MHTs delivering strategies derived from empirically supported psychotherapies may meet user needs and support engagement with effective self-management strategies [34-37].

Because psychotherapy can be considered as a dynamic process that entails ongoing assessment and re-evaluation of an individual’s evolving needs and health status [38-41], MHTs also provide novel opportunities for improving the use of self-management strategies [42-45]. Relative to face-to-face treatment, MHTs can deliver real-time assessments in the context of individuals’ day-to-day life unrestricted by clinical appointment frequency. This assessment information can then be used to adapt the frequency, mode, and content of user support. Tailoring intervention content to individuals has been shown to improve intervention outcomes [46-48], and adaptive interventions that vary intervention options to meet the changing needs of individuals are being investigated [49-51]. These adaptive interventions use fixed decision rules to link assessment of tailoring variables with the delivery of support to provide a flexible intervention design while maintaining replicability [49-51]. Unfortunately, most recently published adaptive intervention protocols have not provided detailed reporting of intervention components required to enable replication [52].

To address the need for increased access to and enhancement of empirically supported tools and content for individuals with bipolar disorder, a novel smartphone-based self-management intervention (LiveWell) has been developed (NCT02405117) and tested in a single-blind randomized controlled trial (NCT03088462). Because adequate description of interventions is essential to facilitate ongoing efforts to improve and disseminate empirically supported treatments [53-56], the adaptive components (decision points, tailoring variables, decision rules, mode, and content of delivery options) for the LiveWell intervention are described here. The overall intervention framework and design for LiveWell, including the delivery and timing of the fixed content and the evaluation methodology, are described in detail elsewhere [57].

## Methods

To assist in developing the expert system providing LiveWell’s adaptive content, an electronic survey (Multimedia Appendix 1) approved by the Northwestern University Institutional Review Board was sent to psychiatrists (N=161) at university-affiliated and private outpatient mental health practices. The survey aimed to obtain psychiatrist feedback on when the smartphone app should prompt users to contact their psychiatrists and when the app should additionally send an email alert to the psychiatrist. In addition, information was requested concerning what type of data would be most useful in a web accessible report for psychiatrists and preferences for being contacted by users. To assist in developing the decision rules, modes and cumulative percent responses were determined from the completed surveys (N=42, 26% response rate). Percent responses were also calculated for preferences and opinions concerning web accessible reports and contact preferences (Multimedia Appendix 2).

To quantify the content of the adaptive delivery options, the behavior change framework that guided the creation of the content and tools for LiveWell was used to code every page of the smartphone app [57]. This framework proposes that (1) engaging in target behaviors improves clinical and recovery outcomes, (2) behavioral determinants govern enactment of target behaviors, and (3) exposure to behavior change technique content and tool use alters behavioral determinants. Determinants and their corresponding techniques are grouped into the following 4 domains: motivational determinants involved in developing an intention to engage in a behavior, volitional determinants involved in enacting the behavior, and environmental determinants and capabilities that impact motivational and volitional processes. This framework provides a means to label app content in terms of outcomes, targets, and determinants addressed by the behavior change techniques delivered on each app page. To quantify variation in the adaptive content delivered to users based on their current assessment data and the decision rules (Multimedia Appendix 3), app content was exported to Excel spreadsheets (Microsoft Corp) for labeling, and the labeled content was processed using custom code written and run using MATLAB (MathWorks) [57].

## Results

### Intervention Overview

The LiveWell intervention aims to decrease episode relapse, reduce symptom burden, and improve quality of life by assisting individuals with managing target behaviors proposed to underlie the impact of existing therapies [57-60]. LiveWell therefore engages users to support managing the signs and symptoms of relapse, taking psychiatric medications as prescribed, obtaining adequate sleep duration, and maintaining regular routines. LiveWell also addresses strengthening social support, managing stressors, and engaging in healthy habits regarding diet, exercise, and substance use.

The intervention consists of technological and human support components including a smartphone app, a secure server and website, and a coach [57-60]. The smartphone app consists of the following 5 primary components: *foundations*, *toolbox*, *wellness plan*, *daily check-in*, and *daily review* [57-60]. The app provides information on bipolar disorder self-management (*foundations*) along with self-assessment surveys and skills content (*toolbox*). The *foundations* and *toolbox* components support developing a personalized *wellness plan* for managing signs and symptoms and maintaining a healthy lifestyle. The core of the intervention is a *daily check-in*, where users monitor medication adherence, sleep duration, routine, and wellness levels. Based on their *daily check-in* data, *daily review* provides adaptive content and directs users to relevant app content in the *foundations*, *toolbox*, and *wellness plan*. A coach provides human support to facilitate app use adherence, self-management strategy use, and communication with mental health providers [58]. The secure server and website provide data summaries and alerts to providers and coaches to facilitate and support clinical care communication (Multimedia Appendix 4).

The LiveWell intervention thus has both fixed and adaptive components. In terms of fixed components, users are asked to attend an initial face-to-face meeting with a coach, complete 6 scheduled coaching calls (weeks 1-4, 6, and 16), read 2 *foundations* lessons per week (weeks 1-4), and use the *daily check-in* each day (weeks 1-16). At an initial face-to-face meeting, a coach helps users identify personalized wellness anchors to assist them in using the *daily check-in’s* wellness rating scale [58,59]. During the scheduled coach calls, users review progress toward their goals and receive guidance on app use (weeks 1-4), develop (week 4) and review (week 6) their personalized wellness plan, and then review their progress and commitment to ongoing use of helpful strategies (week 16). The week 16 scheduled call ends the active phase of the intervention, but users continue to have access to the app and ad hoc coaching support for 48 weeks. While the target behavioral goals and wellness plans are personalized, this personalization is determined by the user in conjunction with the coach without following a predefined algorithm and is thus operator driven and not algorithmically driven [57-60].

The adaptive components of the intervention consist of a rule-based expert system primarily embedded in the *daily check-in* and *daily review* components of the intervention. The *daily check-in* provides the primary user interface for populating the database containing facts about the user’s current status, but data are also collected via a *weekly check-in* consisting of the Patient Health Questionnaire-8 (PHQ8) and Altman Self-Rating Mania (ASRM) scales [61,62]. The knowledge base for the expert system contains the adaptive smartphone app content. It utilizes a clinical status state machine and hierarchical decision tables (if-then/elseif-then) to govern content delivery via the *daily review*. The *daily review* comprises the explanatory system and also serves as the user interface providing adaptive content delivery. The inference engine combines the current data from the user database with the knowledge base to determine the adaptive content to deliver in the moment. In addition to varying the content provided via the *daily review*, the expert system also controls the delivery of smartphone app pop-up messages requesting users to reach out to their clinical care providers by phone. Additionally, the expert system controls the delivery of emails to providers and coaches to stimulate clinical care communication via phone calls, and flags data in daily reports generated for coaches to promote coach clinical reach out and app use adherence phone calls [57-59].

### Decision Points

Decision points are the times when the frequency, mode, or content of the intervention (ie, intervention options) is adapted [49,51,52]. For LiveWell, the decision points occur daily upon user completion of the *daily check-in*, as well as weekly upon completion of the *weekly check-in*. During a pilot study of the intervention, users were initially given the option to complete the *daily check-in* multiple times a day. However, allowing multiple check-ins each day did not appear to elicit user reflection about their wellness status. Instead, multiple check-ins a day seemed to be capturing momentary reactions to daily hassles and uplifts [63]. To encourage users to engage in reflective monitoring rather than in the moment rating, the *daily check-in* was restricted to allow only 1 check-in per day [59]. The app prompts users to check-in each day by delivering an alert in the smartphone’s notification panel. Users can select the time they first receive a daily notification in the app settings. They receive their first notification to check-in at their designated time of the day and then every 2 hours until they check-in or until they receive 3 alerts. The *daily* *review* is only accessible each day after completion of the *daily check-in*. As a result, no adaptive content is delivered via the *daily review* on days when users do not complete the *daily check-in*. Similarly, no adaptive content is delivered if users do not complete the *weekly check-in*.

### Tailoring Variables

Tailoring variables are the values containing information about the user, such as clinical status or health behaviors, which are used to determine the delivery of intervention options [49,51,52]. For LiveWell, the primary tailoring variables are the target behaviors and wellness levels monitored by the *daily check-in* and include users self-reported psychiatric medication adherence (all, some, and none), sleep duration (hours), routine (bedtime/risetime), and wellness rating (0 balanced, −1/+1 daily hassles/uplifts, −2/+2 prodromal/residual symptoms, −3/+3 episode, and −4/+4 crisis). These variables were selected for daily monitoring because they are consistently addressed in the core content of adjunctive psychotherapy interventions [3,16,24,31,64-66] and are readily amenable to goal setting and self-monitoring. LiveWell also contains content addressing building and using supports, managing stressors, and maintaining healthy habits regarding diet, exercise, and substance use [57-60]. However, to simplify the *daily check-in* and make it quick and easy to complete, these additional targets were not included for daily monitoring and use in adapting delivery of intervention options. Because of the importance of clinical status in bipolar disorder, 2 additional tailoring variables were also utilized based on data obtained from the *weekly check-in* (PHQ8 and ASRM scores). In addition, adherence to completing the *daily* and *weekly check-ins* was also used as a tailoring variable.

### Decision Rules

Decision rules link delivery of intervention options and tailoring variables systematically. They include the values of tailoring variables (states, thresholds, and ranges) that determine which intervention option to deliver when and for whom [49,51,52]. LiveWell utilizes algorithm-driven adaptation based on fixed rules to standardize intervention option delivery [52]. In developing the decision rules for LiveWell, it was necessary to consider how variation in medication adherence, sleep duration, routine, and wellness ratings should result in the adaptation of intervention options. In addition, it was also necessary to consider how to rank the importance of variation in each variable. Because pharmacotherapy is the primary treatment for bipolar disorder [3-7], medication adherence was ranked above sleep duration in prioritizing feedback. Sleep duration was then ranked above routine because sleep duration variation is frequently identified as an early warning sign of impending episode relapse, and assisting people with identifying and making plans for managing early warning signs of relapse is an important target of most adjunctive bipolar disorder psychotherapies [67-69]. In fact, many face-to-face bipolar disorder psychotherapy studies have restricted participation to individuals in asymptomatic recovery, and the primary goal of these interventions has been relapse prevention [8-13,16]. However, an important goal of LiveWell is to increase access to self-management techniques derived from empirically supported therapies, so individuals were enrolled in the intervention as long as they were not in a current episode [57].

**Figure 1:**
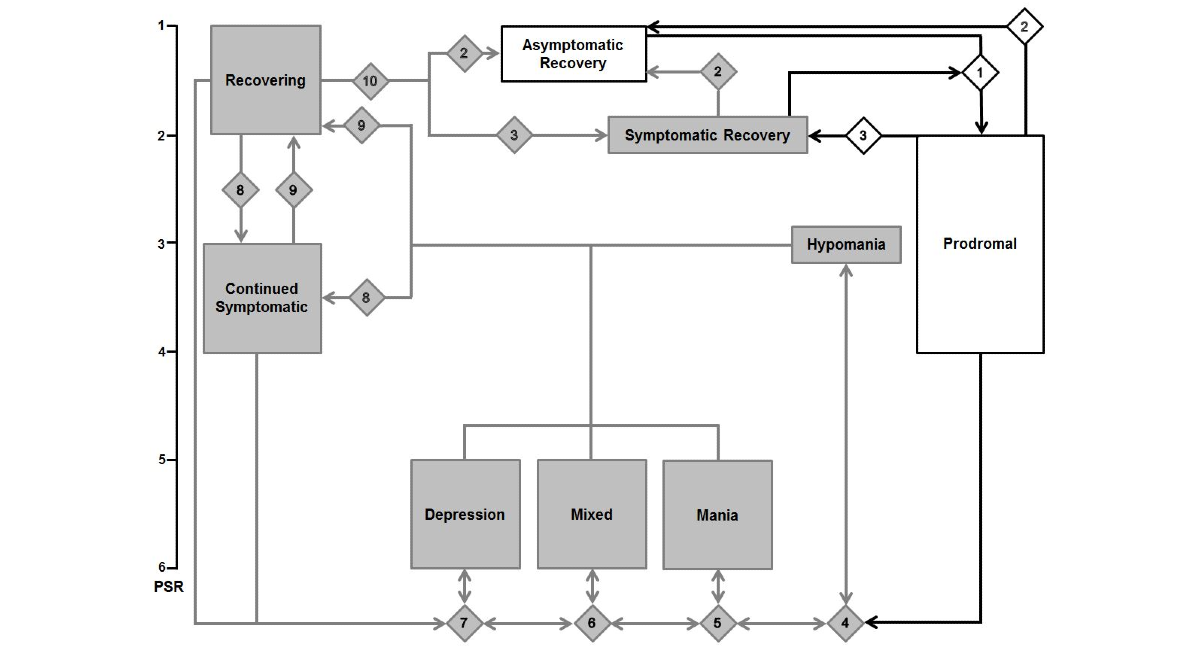
Diagnostic and Statistical Manual of Mental Disorders fourth edition/clinical monitoring form clinical status state machine. Diamonds show decision rule numbers (see Table 1 and Table 2 for rule details). White rectangles, diamonds, and black lines indicate primary clinical status states and transitions addressed by most adjunctive psychotherapies for bipolar disorder and the main focus of the LiveWell intervention. Psychiatric status rating (PSR) is as follows: 1, no symptoms; 1.5, mild symptoms; 2, residual or prodromal symptoms; 3, moderate symptoms; 4, marked symptoms; 5, episode; 6, severe episode.

Because individuals with varying clinical statuses were included initially and people may enter an episode while engaged with LiveWell, clinical status was ranked as the highest priority tailoring variable. This allows the need for substantial adaptation of the treatment approach for individuals with bipolar disorder in various clinical states to be appropriately addressed. As a starting point for developing decision rules to manage clinical status variation, existing criteria for bipolar disorder episodes (mania, hypomania, depression, and mixed) and for nonepisode clinical statuses (asymptomatic and symptomatic recovery, prodromal, continued symptomatic, and recovering) were examined (Table 1 and Table 2). The Diagnostic and Statistical Manual of Mental Disorders fourth edition (DSM4) criteria were used because prior face-to-face psychotherapy trials for bipolar disorder primarily used DSM4 episode criteria [8-16]. They were used for LiveWell’s outcome assessments [57]. Criteria for nonepisode clinical statuses were derived from the clinical monitoring form (CMF) used in a large bipolar disorder intervention study (STEP-BD) where nonepisode clinical statuses were clearly defined [70,71]. These combined criteria were used to define a clinical status state machine where transitions between the 9 possible clinical statuses for bipolar disorder are defined by 10 decision rules derived from DSM4 and the CMF (Figure 1).

The DSM4/CMF state machine highlights the relatively narrow focus of most adjunctive psychotherapies for bipolar disorder (Figure 1). These therapies primarily assist people with remaining in asymptomatic recovery by maintaining medication adherence, adequate sleep duration, and a regular routine, as well as attending to healthy habits and stressors [3,8-13,16,24,31,64,65]. In addition, these therapies assist individuals with recognizing and managing early warning signs and symptoms to avoid transitioning from asymptomatic recovery to a prodromal state and to support transitioning from a prodromal state to asymptomatic recovery rather than entering an episode (Figure 1). This state machine also highlights how entry into an episode can lead to prolonged periods of problems with symptoms; individuals can cycle between episode types, and into and out of continued symptomatic and recovering states during which they are at increased risk for episode relapse [7,72-74]. While this state machine clearly delineates different clinical states and transitions, assessment is complex because of the need to determine the number of symptoms as well as their type and intensity over widely varying durations (4-56 days), and the additional need to assess impairment level and the presence of psychotic symptoms or hospitalization. The DSM4/CMF data necessary to determine the clinical status states for the DSM4/CMF state machine are obtained every 8 weeks during the intervention via telephone assessments delivered by trained assessors blinded to the study arm [57]. However, because the severity of each symptom and the level of impairment due to both symptoms of mania and depression must be assessed, it was decided that this level of assessment via the app would unduly burden the user.

**Table 1:** Diagnostic and Statistical Manual of Mental Disorders fourth edition (DSM4)/clinical monitoring form (CMF): episode criteria.

Clinical status (to)	Entry criteria^a^	Number of moderate symptoms of	Impairment	Consecutive days	DN^b^	PSR^c^
Depression	Yes	Depression ≥5	≥Moderate	≥10/14	7	5-6
Mixed	Yes^d^	Mania and depression^d^	≥Moderate	≥7	6	5-6
Mania	Yes	Mania ≥3 if elevated; mania ≥4 if only irritable	≥Moderate, hospitalized, or psychosis	≥7 or hospitalized	5	5-6
Hypomania	Yes	Mania ≥3 if elevated; mania ≥4 if only irritable	<Moderate, not hospitalized, no psychosis	≥4	4	3

^a^Entry criteria: meeting consecutive day criteria; for mania/hypomania, moderate severity elevated, expansive, or irritable mood; and for depression, moderate severity depressed mood or loss of interest/pleasure. Moderate symptom and impairment criteria based on the clinical monitoring form.

^b^DN: decision rule number.

^c^PSR: psychiatric status rating; 1=no symptoms, 1.5=mild symptoms, 2=residual or prodromal symptoms, 3=moderate symptoms, 4=marked symptoms, 5=episode, and 6=severe episode.

^d^Criteria for mania with concurrent depressive symptoms for 5/7 consecutive days.

**Table 2:** Clinical monitoring form (CMF) clinical status decision rules when not in an episode.

Clinical status (to)	Recovery	Symptom count^a^/impairment	Consecutive days	DN^b^	PSR^c^
Continued symptomatic	No	Symptom count >2 or ≥moderate impairment	≥7^d^	8	3-4
Prodromal	Yes	Symptom count >2 or new^e^ or ≥moderate impairment	≥7^d^	1	2-4
Recovering	No	Symptom count ≤2 and <moderate impairment	≥7^d^ and ≤56	9	1-2
Recovery	Yes	Symptom count ≤2 and <moderate impairment	>56	10	1-2
Symptomatic recovery	Yes	Symptom count >0 but ≤2 and <moderate impairment	≥7^f^	3	2
Asymptomatic recovery	Yes	Symptom count=0 and <moderate impairment	≥7^f^	2	<2

^a^Symptom count: sum of symptom severity. If |severity| ≥1, round up; otherwise, 0. Clinical monitoring form (CMF) symptom severity scale: none=0, mild=0.5, moderate=1, marked=1.5, and severe=2.

^b^DN: decision rule number.

^c^PSR: psychiatric status rating; 1=no symptoms, 1.5=mild symptoms, 2=residual or prodromal symptoms, 3=moderate symptoms, 4=marked symptoms, 5=episode, and 6=severe episode.

^d^The 7 consecutive day window was selected to align with the 7-day evaluation window used for the LIFE-CMF assessments.

^e^Two new moderate, marked, or severe symptoms developed while in recovery.

^f^From prodromal or symptomatic recovery only and not from recovering.

**Figure 2:**
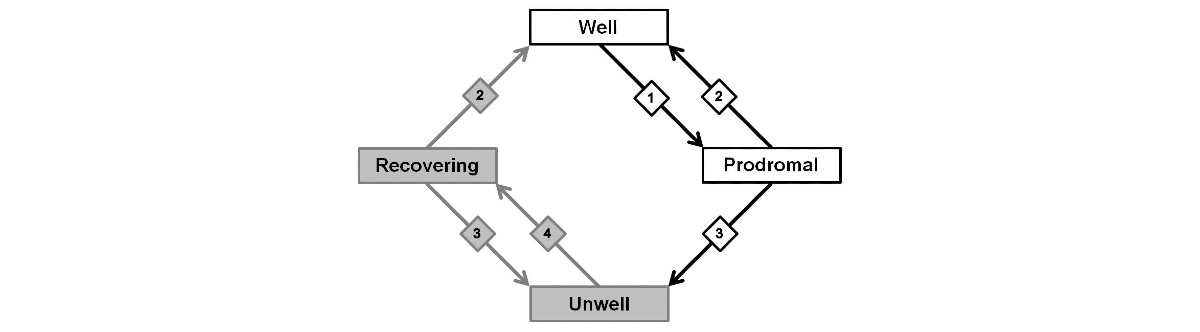
LiveWell clinical status state machine. Diamonds show decision rule numbers (see Table 3 for rule details). White rectangles are the primary states addressed by most adjunctive psychotherapies for bipolar disorder.

Thus, the clinical status state machine for the LiveWell expert system was simplified owing to the complexity of the data required for the DSM4/CMF state machine and the corresponding user assessment burden (Table 3 and Figure 2). Instead of having users rate individual symptom severity, the *daily check-in* uses a personalized 9-point wellness rating scale to allow users to rapidly assess their symptom burden using the *daily check-in* [58,59]. The clinical status assessment window was also limited to users’ last 7 *daily check-ins* to reflect user self-report data covering approximately the prior week depending on *daily check-in* adherence (Table 3). Consistent with the focus of LiveWell on episode relapse prevention, this time window aims to allow relatively rapid adjustment of intervention option delivery with clinical status variation. In addition to limiting the time window, the LiveWell state machine was also simplified by considering only the following 4 clinical states: well, prodromal, unwell, and recovering (Table 3 and Figure 2). Reducing the number of clinical states allows the *daily check-in* wellness ratings to be readily used to identify clinical status state transitions using the same time window for symptoms of depression and mania, thereby reducing the number of decision rules needed and providing more rapid feedback to users when they identify changes in their wellness ratings. In addition, the focus of the LiveWell intervention is on mood episode relapse prevention. The intervention thus aims to assist individuals who have recovered from a mood episode on transitioning from well to prodromal and from prodromal to unwell. If an individual enters a mood episode, the intervention aims to support the individual in working with their providers for treatment. For individuals in the recovering state, the LiveWell intervention adapts techniques developed primarily for mood episode relapse prevention [3,8-13,16,24,31,64,65], with the aim of assisting individuals in transitioning from recovering to well and avoiding transitioning from recovering to unwell. The use of only 4 clinical states for the LiveWell state machine is thus consistent with the focus of the intervention and existing studies from which the intervention content is derived.

**Table 3:** LiveWell clinical status decision rules.

Clinical status (from)	Clinical status (to)	Wellness rating		Decision number
		Criteria	Count^a^	
Well	Prodromal	|WR|^b^ ≥2	≥4	1
Prodromal or recovering	Well	|WR| ≤1	≥5	2
Prodromal or recovering	Unwell	|WR| ≥3	≥5	3
Unwell	Recovering	|WR| ≤2	≥5	4

^a^Count of the last 7 daily check-ins meeting the wellness rating criteria.

^b^|WR|: absolute value of daily check-in wellness ratings.

To aid in defining the thresholds for varying clinical states and to address thresholds for medication adherence and sleep duration, an online electronic survey was sent to practicing psychiatrists to obtain their input on when intervention options should be varied (Multimedia Appendix 1). This questionnaire emphasized identifying the number of days after which psychiatrists felt individuals with bipolar disorder should shift from relying mostly on individual self-management skills and instead engage in self-management via communication and recruiting support from mental health providers. The survey thus asked when the app should deliver a prompt to users requesting that they call their psychiatrist and when the app, via the server, should send an email alert directly notifying the psychiatrist of potential issues (Multimedia Appendix 1). Most psychiatrists indicated that the app should instruct users to call them after 2 to 3 days of experiencing multiple symptoms of mania and after 3 to 4 days of experiencing multiple symptoms of depression or 1 to 2 early warning signs of mania or depression (Multimedia Appendix 2 and Multimedia Appendix 5). In terms of medication adherence, most psychiatrists indicated that the app should instruct users to call them if they reported only taking their psychiatric medications for 3 out of 7 days (43% adherence). For sleep duration, most psychiatrists indicated that the app should instruct users to call them if they reported sleeping less than 4 hours a day for 2 to 3 days and if sleeping less than usual by 3 to 4 hours or more than usual by 4 hours over 7 days. The results were less clear with regard to when psychiatrists would want to receive an email alert (Multimedia Appendix 5), which may be related to most psychiatrists (80%) preferring that their patients contact them via phone rather than via email (Multimedia Appendix 2).

After each completion of the *daily check-in*, the LiveWell inference engine assesses the user’s current clinical status and last 7 *daily check-in* wellness ratings and applies the knowledge base rules to update the user’s clinical status (Table 3 and Figure 2). The user’s initial clinical status is set by the coach using baseline study assessment data and is then updated after each check in. For example, if a user’s clinical status is well and the absolute value of their wellness ratings are 2 or greater for 4 or more of the last 7 check-ins, the user’s clinical status is updated to prodromal (decision number 1 in Table 3, Figure 2). The coach assists users in understanding and identifying personalized anchors indicating that a wellness rating of +2 or −2 corresponds to the presence of early warning signs or symptoms when doing well or some residual symptoms when improving from being in an episode [58]. The threshold of 4 check-ins corresponds to the DSM4 criteria for hypomania (Table 1) and to psychiatrist feedback from the survey indicating psychiatrists would like their patients to contact them when experiencing manic or depressive symptoms or early warning signs (Multimedia Appendix 5). For the transition from prodromal or recovering to unwell, a count of 5 or more for the last 7 check-ins with an absolute wellness rating value of 3 or greater was used (decision number 3 in Table 3, Figure 2). A wellness rating of 3 is defined for users as corresponding to multiple symptoms day to day, with some impairment likely indicating a mood episode. The threshold of 5 counts out of 7 corresponds to the CMF threshold for a depressive episode using DSM4 criteria (Table 1), which captures the DSM4/CMF criteria of depressive symptoms being present most days for 2 weeks (ie, 10/14 days). However, for the LiveWell clinical status state machine, the count has been reduced by half to reflect the approximately 1 week window of the state machine (ie, 5/7 days).

**Figure 3:**
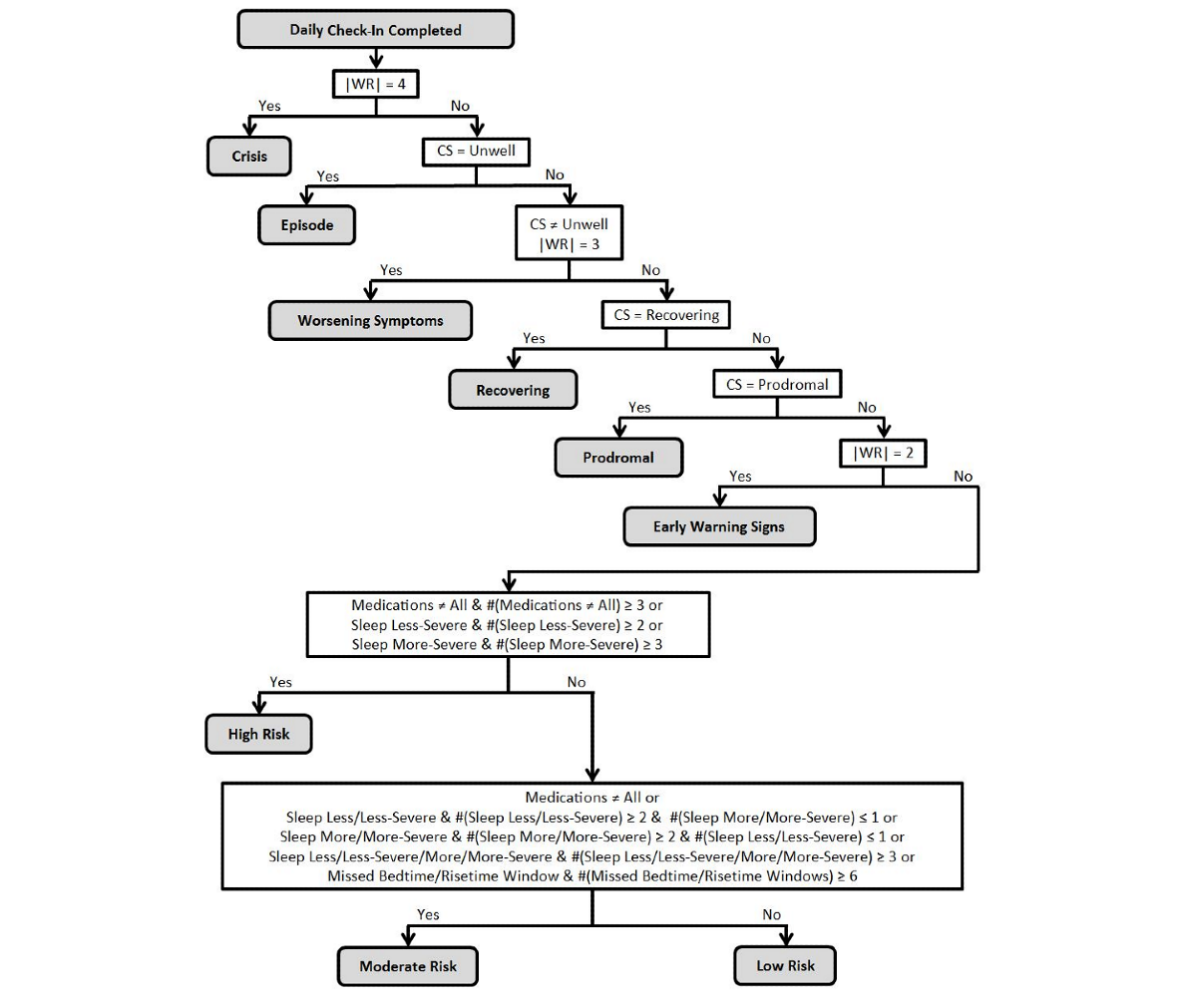
Overview of the daily review decision rules. Only primary decisions are displayed for complete decision rules (see Multimedia Appendix 3 and Multimedia Appendix 6). #(X) indicates the count of the last 4 daily check-ins satisfying the condition. Sleep Less-Severe indicates ≤4 hours of sleep. Sleep More-Severe indicates ≥12 hours of sleep or ≥personalized goal upper limit plus 4 hours, whichever is less. Sleep Less indicates <personalized goal lower limit (eg, 6 hours). Sleep More indicates >personalized goal upper limit (eg, 8 hours). Bedtime Window is the personalized 1.5-hour window for going to bed (eg, 10:30 PM to midnight). Risetime Window is the personalized 1.5-hour window for getting up to start the day (eg 7:00 to 8:30 AM). CS: clinical status; |WR|: absolute value of wellness rating.

After the state machine determines clinical status, a hierarchical decision table is used to decide which of the 26 content categories should be delivered by the *daily review* (Figure 3, Multimedia Appendix 3, and Multimedia Appendix 6) [57]. Because clinical status and wellness ratings are prioritized, the *daily review* decision table starts by examining these variables and proceeds using an if-then/elseif-then decision tree. Thus, if a user enters a wellness rating of +4 or −4, the crisis content categories severe up or down are selected. After these 2 categories, the next 15 content categories are selected by examining the clinical status and the current wellness rating (Multimedia Appendix 3 and Multimedia Appendix 6). These content categories address continuing or improving in the clinical states unwell, prodromal, and recovering, as well as worsening symptoms (wellness ratings +3 or −3) while not in the unwell state (well, prodromal, and recovering), and the presence of early warning signs (wellness ratings +2 or −2) while in the well state (Figure 3). The decision rules for the remaining 9 categories examine user self-report data regarding medication adherence, sleep duration, and routine using data from the 4 last check-ins (Figure 3). This approximately 4-day window for deciding when to deliver content addressing these targets was selected based on the psychiatrist survey feedback (Multimedia Appendix 5).

In addition to varying the content of the *daily review*, the LiveWell expert system also utilizes separate decision tables to prompt users to communicate with their psychiatrists by phone and to deliver emails to activate coaches and enrolled mental health providers to reach out to users via phone (Multimedia Appendix 3 and Multimedia Appendix 7). For *daily check-in* data, a hierarchical decision table organizes selection of clinical reach out messages based on the presence of crisis wellness ratings (+4 or −4), transition to unwell or prodromal states, worsening symptoms when not unwell, or more severe problems with medication adherence and sleep duration (Multimedia Appendix 7). The *daily check-in* clinical reach out decision table thresholds are primarily derived from the psychiatrist survey feedback. A separate set of decision rules trigger clinical reach outs after the *weekly check-in* if the user’s score indicates new onset of a manic or depressive episode (Multimedia Appendix 7). The thresholds for the *weekly check-in* are set based on the published thresholds indicating the possible presence of manic or depressive episodes for the ASRM and PHQ8 scales [61,62]. The psychiatrist survey feedback indicated that most psychiatrists felt a higher threshold might be used (Multimedia Appendix 2), but the published thresholds were selected based on the request of the Northwestern University Institutional Review Board. In a report generated for coaches each day (Multimedia Appendix 8), an additional set of decision rules also flags data indicating problems with *daily* or *weekly check-in* adherence (Multimedia Appendix 7).

### Intervention Options

**Figure 4:**
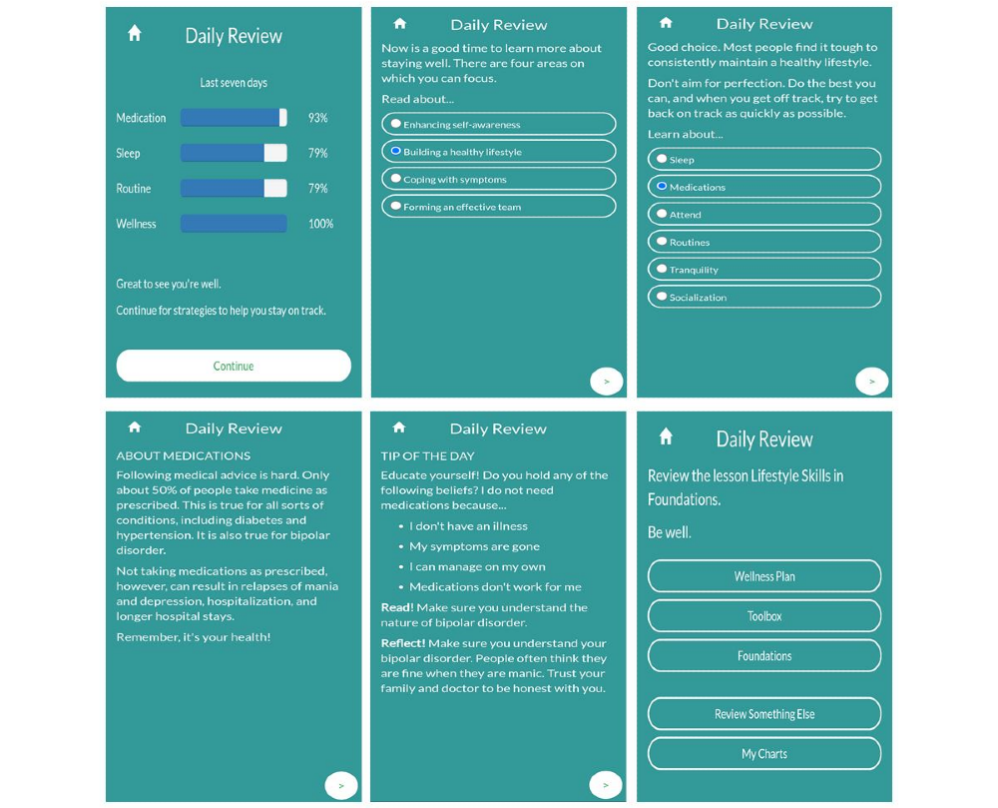
Daily review example for the low-risk content category.

Intervention options include variation in intervention frequency, mode, or content implemented at the decision points following the decision rules [49,51,52]. For LiveWell, the *daily review* provides the primary user interface and explanatory system delivering adaptive content to the user. The *daily review* utilizes a fixed format of 3 to 6 app display pages to adaptively present content for the 26 categories determined by the *daily review* decision rules (Multimedia Appendix 3 and Multimedia Appendix 6) [57]. The first page of the *daily review* provides brief feedback about the content category being addressed and the user data indicating why that content category is deemed relevant (Figure 4). In addition, the first page of the *daily review* provides visual feedback regarding target goal achievement using bar graphs to display the percent of days over the last 7 days that the user met the goal for each target. The *daily review* then delivers additional pages of content addressing the selected category with 1 to 4 pages presented depending on the category (Multimedia Appendix 3). The final page of the *daily review* then reiterates the content category being addressed and provides suggestions and links to other sections of the app for review of the selected content category (Figure 4).

**Figure 5:**
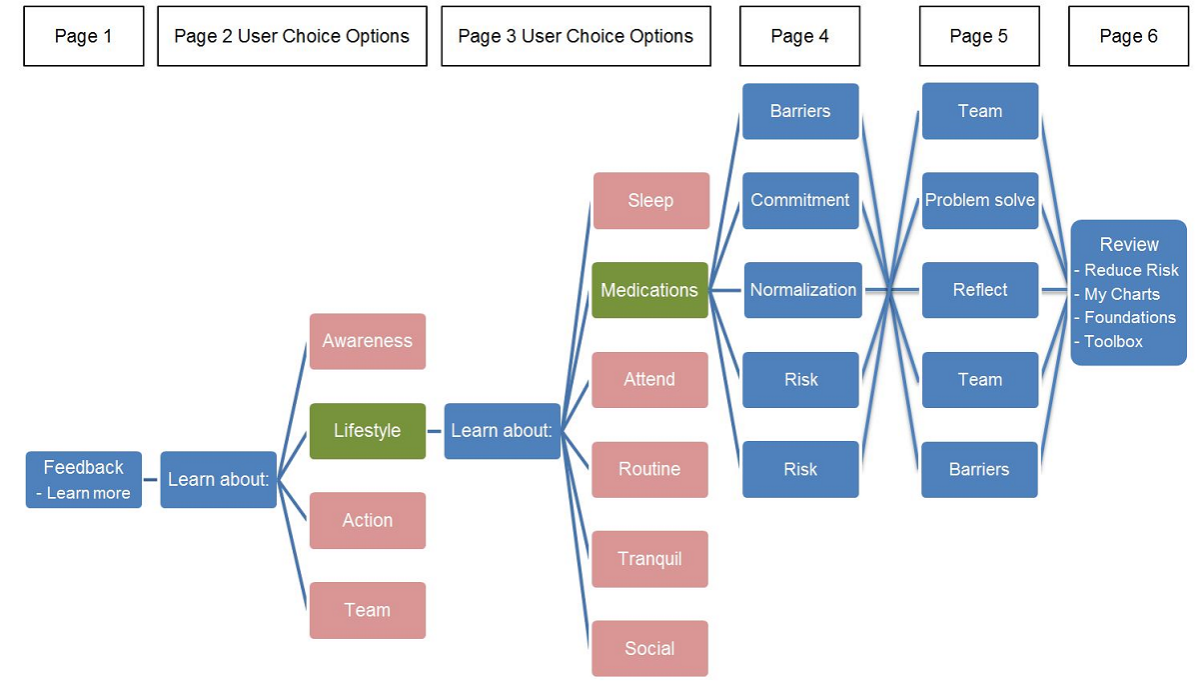
Daily review flowsheet for the low-risk content category.

To reduce the potential for user fatigue when viewing the same content category multiple times, the first and last pages of the *daily review* randomly present one of a number of pages providing similar content stated in varying ways (Figures 4 and 5). In addition, for pages 2 to 5 of the *daily review*, a given page number (eg, page 2) may have multiple permutations of similar content or multiple unique pages of content that are randomly selected for presentation each time a user views a content category [57]. In some cases, a unique page of content (page 2) is linked to a subsequent unique page of content (page 3) so that only the prior page is randomly selected, but in other cases, subsequent unique pages are randomly selected. Randomly combining permuted and unique pages of content addressing a selected category allows the *daily review* to vary provided content about a given category over multiple views.

The content of each page is typically varied by providing information or suggesting tools to address different motivational, volitional, environmental, or capability-based determinants relevant to the selected category (Figure 5). To provide additional personalization with the aim of maintaining user engagement, some of the *daily review* content categories also present pages where users can select choice options which then determine the content of the subsequent *daily review* page. For instance, if the low-risk category is selected by the inference engine, then page 2 of the *daily review* allows the user to learn more about enhancing self-awareness, building a healthy lifestyle, coping with symptoms, or forming an effective team to assist with staying well (Figure 5). If the user selects lifestyle on page 2, then page 3 allows the user to select 1 of 6 categories (sleep, medication, attend, routine, tranquil, and social) to learn more about supporting different aspects of a healthy lifestyle while living with bipolar disorder.

Although 26 content categories are adaptively delivered via the *daily review*, the content as a whole can be grouped into the following 3 tiers: (1) low, moderate, and high risk categories where the user’s clinical status is well and early warning signs or worsening symptoms are not endorsed; (2) categories where the user’s clinical status is well and early warning signs or worsening symptoms are endorsed or where the user’s clinical status is prodromal or recovering; and (3) categories where the user is in an episode or crisis (Figure 3). The first-tier categories contain most of the *daily review* content (71% of available pages), and their content addresses the targets sleep duration, medication adherence, managing signs and symptoms, building support, and keeping a regular routine (30%, 22%, 20%, 9%, and 8% of tier content, respectively) and the following determinants underlying engagement in these target behaviors: attitudes and perceptions, knowledge, support, evaluation, and planning (18%, 15%, 13%, 11%, and 10% of tier content, respectively). For the second-tier categories, the *daily review* content (22% of available pages) addresses the targets managing signs and symptoms and using supports (86% and 6% of tier content, respectively) and the following determinants: evaluation, skills, knowledge, adjustment, and practice (22%, 16%, 16%, 10%, and 7% of tier content, respectively). For the third-tier categories, the *daily review* content (7% of available pages) only addresses managing signs and symptoms (99% of tier content) and the following determinants: evaluation, skills, adjustment, knowledge, and support (22%, 18%, 16%, 15%, and 13% of tier content, respectively). Thus, as users move up the tiers to more acute content categories, the focus of the content shifts to managing signs and symptoms and utilizing supports (Multimedia Appendix 3). In fact, the crisis categories triggered by user entry of a wellness rating of +4 or −4 are the only categories that do not follow the general format of *daily review* content delivery. For these 2 categories, a single page of content without bar graphs is presented requesting that users take immediate action and contact their psychiatrists and supports, and if in danger of self-harm, call 911 or go to the nearest emergency room.

**Figure 6:**
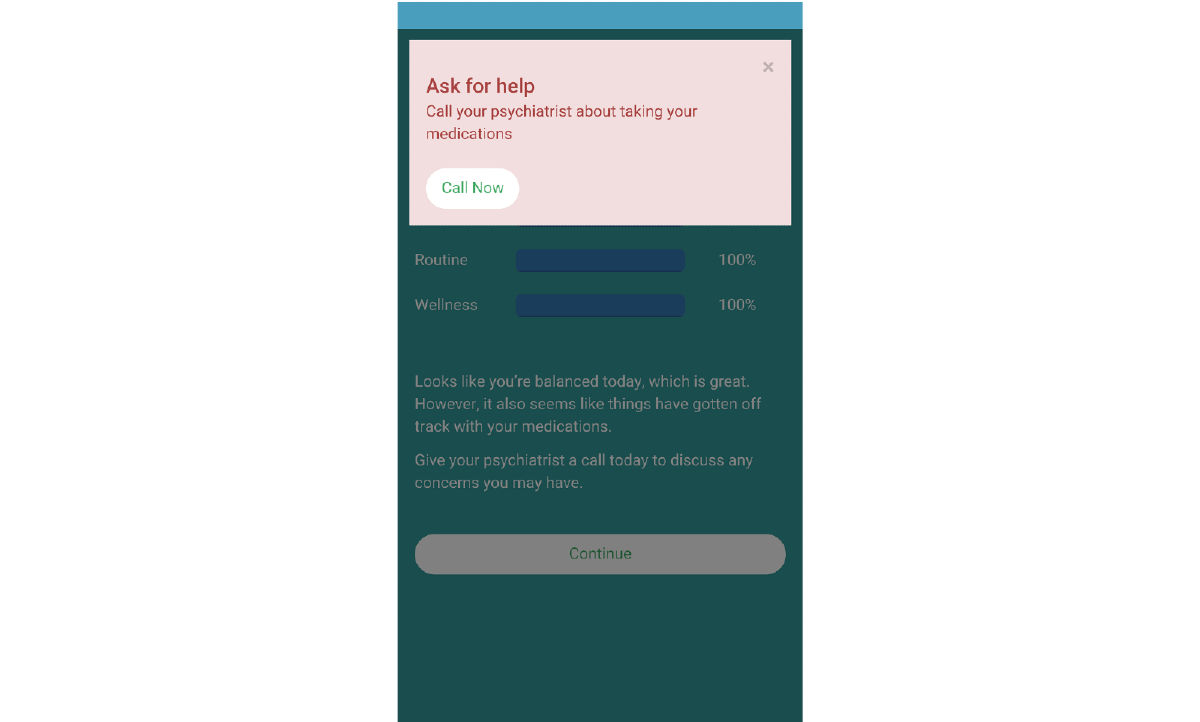
Daily review pop-up.

The expert system also prompts clinical care communication to reinforce the importance of engaging supports when having difficulties (Multimedia Appendix 3 and Multimedia Appendix 7). When these decision rules activate a clinical reach out, a pop-up is provided to the user asking the user to call their psychiatrist to address the selected category. To facilitate the user making this call, the pop-up includes a button linked to the psychiatrist’s phone number (Figure 6). In addition, an email is sent to the coach and enrolled providers alerting them to the situation (Multimedia Appendix 3). The coach acts on these alerts following structured protocols described in detail elsewhere [58]. Finally, when user adherence with completing *daily* and *weekly check-ins* falls below predetermined thresholds (Multimedia Appendix 7), coaches are also prompted via flagged data in the daily coaching reports (Multimedia Appendix 8). Coaches then follow-up with phone calls, texts, or emails to address adherence issues using a motivational interviewing approach [58]. Overall, the clinical reach out rules aim to shift the mode of intervention delivery from self-management using individual skills to engaging in self-management via effective communication and elicitation of support from mental health care providers and other supports.

## Discussion

The expert system for adaptively delivering content and facilitating clinical care communication for LiveWell is described in detail in this paper to enable replication and ongoing improvement of adaptive MHTs. The LiveWell intervention aims to assist individuals with bipolar disorder in using self-management strategies to manage targets proposed to reduce relapse risk and symptom burden, and improve quality of life [59]. Information from empirically supported psychotherapies for bipolar disorder, health psychology behavior change theories, and chronic disease self-management models directed the selection of the tailoring variables, creation of intervention options, and development of decision rules [59]. User-centered design information guided the timing of the decision points [59]. Feedback from an electronic survey of psychiatrists was combined with existing bipolar disorder literature to define the values of the tailoring variable used in the decision rules.

Because adjunctive psychotherapies for bipolar disorder encourage individuals to manage multiple targets, it was also necessary to develop a hierarchy of tailoring variables ordered as follows: (1) managing signs and symptoms, (2) medication adherence, (3) sleep duration, and (4) routine. A simplified clinical status state machine was developed to predict clinical status based on self-reported wellness rating data. Clinical status from this state machine was then incorporated into hierarchal decision tables (if-then/elseif-then) that select content for users and alerts for providers. For each target behavior selected, intervention options address a variety of determinants proposed to govern engagement in target behaviors, including attitudes and perceptions, knowledge, support, evaluation, planning, skills, adjustment, and practice [57]. When providing adaptive content, the user interface (*daily review*) randomly varies information regarding different determinants and provides user choice options with the goal of reducing user fatigue when category content is delivered multiple times.

There are limitations to the expert system currently developed for the LiveWell intervention. While simplifying the clinical status state machine by using a 9-point wellness rating scale reduces the burden on the user in completing the *daily check-in*, the relationship between the clinical status states determined using these data and those determined by DSM4/CMF outcome assessments has not yet been identified. In addition, while feedback from providers was used to establish the thresholds for adaptive delivery of intervention content, there was significant variation in provider responses related to clinical status, medication adherence, and sleep duration. In their comments, providers also noted that these thresholds may vary between individuals and over time, and that the importance of different targets may also vary for different individuals (Multimedia Appendix 2). Additional data will thus be needed to assess how to optimize the hierarchical organization of feedback delivery and the thresholds for delivering this feedback. Despite these limitations, we hope that the comprehensive description of the expert system delivering the adaptive content for LiveWell and the underlying design decisions addressed during the development of this system will facilitate the ability to replicate, improve, implement, and disseminate effective adaptive MHTs for bipolar disorder and other mental health conditions.

## Appendix

Multimedia Appendix 1 LiveWell psychiatrist survey.

Multimedia Appendix 2 LiveWell psychiatrist survey data.

Multimedia Appendix 3 LiveWell daily review logic and content.

Multimedia Appendix 4 Dashboard report examples.

Multimedia Appendix 5 Psychiatrist survey summary.

Multimedia Appendix 6 Daily review decision rules.

Multimedia Appendix 7 Clinical and adherence reach out decision rules.

Multimedia Appendix 8 Coach report examples.

## Abbreviations

ASRM: Altman Self-Rating Mania
CMF: clinical monitoring form
DSM4: Diagnostic and Statistical Manual of Mental Disorders fourth edition
MHT: mental health technology
PHQ8: Patient Health Questionnaire-8
