# Supplementary material for: A Smartphone-Based Self-management Intervention for Individuals With Bipolar Disorder (LiveWell): Protocol Development for an Expert System to Provide Adaptive User Feedback
Source: JMIR Form Res. 2021 Dec 24;5(12):e32932. doi: 10.2196/32932 (PMC8742209; doi:10.2196/32932)
Supplement: Multimedia Appendix 1 [file formative_v5i12e32932_app1.pdf]

Dear provider,

You are receiving this research survey link because of your affiliation with the Department of Psychiatry and Behavioral Sciences of the Feinberg School of Medicine at Northwestern University.

As part of a research project funded by the National Institutes of Mental Health, we are developing a smartphone application for use by patient's with bipolar 1 disorder. The application will provide patients with access to standard psychosocial lessons for the management of bipolar disorder. It will also collect self-report and other data to provide immediate feedback to patients about their risk and symptom status via the phone and to update psychiatrists via email and a secure web portal.

We are asking if you would take 5 to 10 minutes of your time to complete our survey. Your input will help us to design a more effective application that we hope will assist patients and psychiatrists in the management of bipolar disorder.

Responses will be anonymous and confidential.

If you have any questions, please contact Dr. Evan Goulding at 312-503-1189.

We appreciate your time and effort in completing this survey. Thank you.

Sincerely,

Evan Goulding, MD, PhD
Assistant Professor
Department of Psychiatry and Behavioral Sciences
Feinberg School of Medicine
Northwestern University

LiveWell Physician Survey

Thank you for agreeing to help us improve our development of a smartphone application for bipolar disorder.

We are conducting this survey to obtain your input in several important areas of the application's design including:

1) When the application should recommend a patient contact his/her psychiatrist

2) When the application should additionally send an email alert to the psychiatrist

3) What data would be most useful in a web accessible report for psychiatrists.

Additionally, we want to understand your thoughts about how this type of application might be most helpful to you and your patients. We also want to identify barriers to its use and usefulness.

Before you begin the survey, please read over this brief description of who will be participating and what they will be doing.

Participating population:

- Patients with bipolar 1 disorder
- 2 acute mood episodes in the last 2 years
- In remission at enrollment (≥ 8weeks with ≤ 2 symptoms)
- Not meeting criteria for a substance use disorder in the last year
- In treatment with a psychiatrist
- Patient's psychiatrist willing to enroll to receive email alerts and patient status reports via a web portal.

LiveWell Program:

The LiveWell application will provide brief standard psychosocial lessons on the phone. These lessons will cover: basic facts about bipolar disorder, the importance of medications, lifestyle skills to reduce risk, coping skills to manage symptoms, and developing a support system.

The lessons will help patients create a personalized wellness plan focused on being aware of how they are doing and taking action to stay well using medications, skills, and supports. A coach will train the patient to use the application and will be available (by phone and email) to assist the patient in getting the most out of the application.

A central feature of the application is a daily check in and review. Using the phone, participants will check-in daily and record their sleep time, medication use, rate their wellness and the presence of any early warning signs. In addition, sensors on the phone and a wrist-worn actimeter will obtain additional behavioral data including daily patterns of sleep, activity and social routines. According to their self-report and sensor data, participants will receive feedback in the daily review that will direct their attention to areas of concern (medications, sleep, routine, early warning signs, wellness) and guide them towards solutions.

If there are indications that a mood episode may be developing or is present, patients will receive feedback recommending that they call their psychiatrist. Depending on the situation and input from participating psychiatrists, the application may also send the patient's psychiatrist an email alert.

If there are indications of severe symptoms, patients will be instructed to call 911 or go to the emergency room. Study staff will receive an email alert and will contact the patient and psychiatrist.

The LiveWell application should instruct your patient to CALL you,

1. If your patient is not in a crisis but reports multiple symptoms of mania for how many days:

◯ 1
◯ 2
◯ 3
◯ 4
◯ None of the above

Comments:

____

2. If your patient is not in a crisis but reports multiple symptoms of depression without suicidal ideation for how many days:

◯ 1
◯ 2
◯ 3
◯ 4
◯ None of the above

Comments:

____

The LiveWell application should instruct your patient to CALL you and the LiveWell application should email you an URGENT ALERT,

3. If your patient is not in a crisis but reports multiple symptoms of mania for how many days:

◯ 1
◯ 2
◯ 3
◯ 4
◯ None of the above

Comments:

____

4. If your patient is not in a crisis but reports multiple symptoms of depression without suicidal ideation for how many days:

◯ 1
◯ 2
◯ 3
◯ 4
◯ None of the above

Comments:

____

The LiveWell application should instruct your patient to CALL you,

5. If your patient exhibits 1-2 early warning signs for how many days:

◯ 1
◯ 2
◯ 3
◯ 4
◯ None of the above

Comments:

____

The LiveWell application should instruct your patient to CALL you and the LiveWell application should email you an URGENT ALERT,

6. If your patient exhibits 1-2 early warning signs for how many days:

◯ 1
◯ 2
◯ 3
◯ 4
◯ None of the above

Comments: ____

The LiveWell application should instruct your patient to CALL you,

7. If over 14 days, your patient on average, takes his medications:

◯ 86% (12 of 14 days)
◯ 71% (10 of 14 days)
◯ 50% (7 of 14 days)
◯ 28% (4 of 14 days)
◯ None of the above

Comments: ____

8. If over 7 days, your patient on average, takes his medications :

◯ 86% (6 of 7 days)
◯ 71% (5 of 7 days)
◯ 57% (4 of 7 days)
◯ 43% (3 of 7 days)
◯ None of the above

Comments: ____

9. If over 4 days, your patient on average takes his medications:

◯ 75% (3 of 4 days)
◯ 50% (2 of 4 days)
◯ 25% (1 of 4 days)
◯ 0% (0 of 4 days)
◯ None of the above

Comments: ____

The LiveWell application should instruct your patient to CALL you and the LiveWell application should email you an URGENT ALERT,

10. If over 14 days, your patient on average, takes his medications:

◯ 86% (12 of 14 days)
◯ 71% (10 of 14 days)
◯ 50% (7 of 14 days)
◯ 28% (4 of 14 days)
◯ None of the above

Comments: ____

11. If over 7 days, your patient on average, takes his medications:

◯ 86% (6 of 7 days)
◯ 71% (5 of 7 days)
◯ 57% (4 of 7 days)
◯ 43% (3 of 7 days)
◯ None of the above

Comments:

____

12. If over 4 days, your patient on average takes his medications:

◯ 75% (3 of 4 days)
◯ 50% (2 of 4 days)
◯ 25% (1 of 4 days)
◯ 0% (0 of 4 days)
◯ None of the above

Comments:

____

The LiveWell application should instruct your patient to CALL you,

13. If your patient sleeps only 0-2 hours for how many days:

◯ 1
◯ 2
◯ 3
◯ 4
◯ None of the above

Comments:

____

14. If your patient sleeps only 2-4 hours for how many days:

◯ 1
◯ 2
◯ 3
◯ 4
◯ None of the above

Comments:

____

The LiveWell application should instruct your patient to CALL you and the LiveWell application should email you an URGENT ALERT,

15. If your patient sleeps only 0-2 hours for how many days:

◯ 1
◯ 2
◯ 3
◯ 4
◯ None of the above

Comments:

____

16. If your patient sleeps only 2-4 hours for how many days:

- ◯ 1
- ◯ 2
- ◯ 3
- ◯ 4
- ◯ None of the above

Comments:

____

The LiveWell application should instruct your patient to CALL you,

17. If over 7 days, your patient's average sleep duration is LESS than usual by how many hours?

- ◯ 1
- ◯ 2
- ◯ 3
- ◯ 4
- ◯ None of the above

Comments:

____

18. If over 3 days, your patient's average sleep duration is LESS than usual by how many hours?

- ◯ 1
- ◯ 2
- ◯ 3
- ◯ 4
- ◯ None of the above

Comments:

____

19. If over 1 day, your patient's sleep duration is LESS than usual by how many hours?

- ◯ 1
- ◯ 2
- ◯ 3
- ◯ 4
- ◯ None of the above

Comments:

____

20. If over 7 days, your patient's average sleep duration is MORE than usual by how many hours?

- ◯ 1
- ◯ 2
- ◯ 3
- ◯ 4
- ◯ None of the above

Comments:

____

21. If over 3 days, your patient's average sleep duration is MORE than usual by how many hours?

- ◯ 1
- ◯ 2
- ◯ 3
- ◯ 4
- ◯ None of the above

Comments: ____

22. If over 1 day, your patient's sleep duration is MORE than usual by how many hours?
- ◯ 1
- ◯ 2
- ◯ 3
- ◯ 4
- ◯ None of the above

Comments: ____

The LiveWell application should instruct your patient to CALL you and the LiveWell application should email you an URGENT ALERT,

23. If over 7 days, your patient's average sleep duration is LESS than usual by how many hours?
- ◯ 1
- ◯ 2
- ◯ 4
- ◯ 5
- ◯ None of the above

Comments: ____

24. If over 3 days, your patient's average sleep duration is LESS than usual by how many hours?
- ◯ 1
- ◯ 2
- ◯ 3
- ◯ 4
- ◯ None of the above

Comments: ____

25. If over 1 day, your patient's sleep duration is LESS than usual by how many hours?
- ◯ 1
- ◯ 2
- ◯ 3
- ◯ 4
- ◯ None of the above

Comments: ____

26. If over 7 days, your patient's average sleep duration is MORE than usual by how many hours?
- ◯ 1
- ◯ 2
- ◯ 3
- ◯ 4
- ◯ None of the above

Comments: ____

27. If over 3 days, your patient's average sleep duration is MORE than usual by how many hours?

◯ 1
◯ 2
◯ 3
◯ 4
◯ None of the above

Comments:

____

28. If over 1 day, your patient's sleep duration is MORE than usual by how many hours?

◯ 1
◯ 2
◯ 3
◯ 4
◯ None of the above

Comments:

____

The LiveWell application should instruct your patient to CALL you,

29. If your patient's weekly Physician Health Questionnaire 8 (PHQ8, 0-24, ≥ 10 depression likely) score was?

◯ 5
◯ 10
◯ 15
◯ 20
◯ None of the above

Comments:

____

30. If your patient's weekly PHQ8 score was greater than usual by how many points?

◯ 4
◯ 5
◯ 6
◯ 7
◯ None of the above

Comments:

____

31. If your patient's weekly Altman Self-Rating Mania Scale (ASRM, 0-20, ≥ 6 mania likely) score was

◯ 4
◯ 6
◯ 10
◯ 16
◯ None of the above

Comments:

____

32. If your patient's ASRM score was greater than usual by how many points:

◯ 4
◯ 5
◯ 6
◯ 7
◯ None of the above

Comments:

____

The LiveWell application should instruct your patient to CALL you and the LiveWell application should email you an URGENT ALERT,

33. If your patient's Physician Health Questionnaire 8 (PHQ8) score was:

◯ 5
◯ 10
◯ 15
◯ 20
◯ None of the above

Comments:

____

34. If your patient's PHQ8 score was greater than usual by how many points:

◯ 6
◯ 7
◯ 8
◯ 9
◯ None of the above

Comments:

____

35. If your patient's Altman Self-Rating Mania Scale (ASRM) score was:

◯ 6
◯ 10
◯ 14
◯ 16
◯ None of the above

Comments:

____

36. If your patient's ASRM score was greater than usual by how many points:

◯ 4
◯ 5
◯ 6
◯ 7
◯ None of the above

Comments:

____

Each provider will have access via a secure web portal to a report summarizing their patient's data summarized by week. The following questions will help us learn what you, as a provider would like included in the report.

LiveWell Physician Report

[Attachment: "LiveWell Physician Report Mock-Up.pdf"]

37. In regards to sleep, would you like the report to include your patient's weekly average:

- ☐ Duration
- ☐ Latency
- ☐ Efficiency (total sleep time/time in bed)
- ☐ Percent nocturnal
- ☐ None of the above

(May select more than one.)

Comments: ______________________________

38. In regards to routine, would you like the report to include your patient's weekly average:

- ☐ Percent time spent at home
- ☐ Number of locations your patient visits
- ☐ Number of phone calls your patient makes/receives
- ☐ Number of texts your patient makes/receives
- ☐ A composite index summarizing the regularity of patient's daily routine

(May select more than one.)

39. In addition to a report summarizing weekly data, would you like access to a report with daily data?

- ◯ Yes
- ◯ No
- ◯ No preference

Comments: ______________________________

40. In addition to viewing the data in a table, would you like to be able to view the data as a graph?

- ◯ Yes
- ◯ No
- ◯ No preference

Comments: ______________________________

Please comment on the following:

41. Do you have regular internet access at your office?

- ◯ Yes
- ◯ No

42. Do you check your email on a daily basis?

- ◯ Yes
- ◯ No

43. Are you comfortable with the LiveWell application instructing your patient to contact you?

- ◯ Yes
- ◯ No

Comments: ______________________________

44. How do you usually prefer your patients contact you?	◯ Phone<br>◯ Email<br>◯ Pager<br>◯ Other
Comments:	__________________________
45. How useful do you think this application could be to your patients with bipolar 1 disorder?	◯ Very helpful<br>◯ Somewhat helpful<br>◯ Neither helpful nor harmful<br>◯ Somewhat harmful<br>◯ Very harmful
Comments:	__________________________
46. How useful do you think this application could be to you as a provider?	◯ Very helpful<br>◯ Somewhat helpful<br>◯ Neither helpful nor harmful<br>◯ Somewhat harmful<br>◯ Very harmful
Comments:	__________________________
47. Do you think having a patient use this application would be a burden for you?	◯ Yes<br>◯ No
Comments:	__________________________
Any Additional Comments:	__________________________
