# Supplementary material for: A Smartphone-Based Self-management Intervention for Individuals With Bipolar Disorder (LiveWell): Protocol Development for an Expert System to Provide Adaptive User Feedback
Source: JMIR Form Res. 2021 Dec 24;5(12):e32932. doi: 10.2196/32932 (PMC8742209; doi:10.2196/32932)
Supplement: Multimedia Appendix 4 [file formative_v5i12e32932_app4.pdf]

LiveWell Clinical Status Summary

Example User 1

Daily Summary for July 29, 2018

Clinical Status: Well (1)

Recommendation: Try sleeping about the same duration each night. Use lifestyle skills to help.

	Daily Check In	Goal
**Medication:**	Took All	Take All
**Sleep Duration:**	6 hours	7 - 9 hours
**Bedtime:**	12:30 AM	9:30 PM - 11:00 PM
**Risetime:**	6:30 AM	3:00 AM - 5:00 AM
**Wellness Rating:**	Balanced (0)	-1 to +1

Weekly Summary

Domains	Metric		07/23/18	07/16/18	07/09/18	07/02/18	06/25/18	06/18/18	06/11/18	06/04/18	05/28/18	05/21/18	05/14/18	05/07/18	04/30/18
Weekly Check In Mania	Survey(AMRS) >=6 possible mania		0	0	0	0	0	0	0	0	0	0	0	4	3
	Early Warning Signs, 8 maximum		0	0	0	0	0	0	0	0	0	0	0	1	0
Weekly Check In Depression	Survey(PHQ8) >=10 possible depression		6	2	5	0	0	0	2	3	1	4	6	10	7
	Early Warning Signs, 8 maximum		0	0	0	0	0	0	0	0	0	1	0	2	2
Daily Check Ins	Days Reported, 7 maximum		7	7	7	7	7	7	7	7	7	7	7	7	4
Sleep	Duration Min, hours		5	3	3	2	3.5	0	0	0	2.5	5	0	0	5
	Duration Mean, hours		7.5	5.5	7	7	6	4.5	4.5	5.5	7	7	6	4.5	5.5
	Duration Max, hours		11	10	9	10	10	8	9	9	9	9	8.5	6.5	6
	In Range, %		14	29	71	57	14	29	29	43	71	71	57	0	0
Routine	In Bedtime Window, %		0	14	0	14	0	29	14	0	0	0	29	29	0
	In Risetime Window, %		14	57	71	57	71	29	43	43	43	57	57	57	50
Medications	Adherence, %		100	100	100	100	100	100	100	100	100	100	100	100	100
Wellness	Moderate Up, %	(+3, Episode)	0	0	0	0	0	0	0	0	0	0	0	0	0
	Mild Up, %	(+2, Prodromal)	0	0	0	0	0	0	14	0	0	0	0	0	0
	Well, %	(-1 to +1, Balanced)	100	100	71	100	100	100	86	100	100	100	86	57	100
	Mild Down, %	(-2, Prodromal)	0	0	29	0	0	0	0	0	0	0	14	43	0
	Moderate Down, %	(-3, Episode)	0	0	0	0	0	0	0	0	0	0	0	0	0

LiveWell Clinical Status Summary

Example User 2

Daily Summary for June 23, 2019

Clinical Status: Well (1)

Recommendation: Try sleeping more. Use lifestyle skills to help.

	Daily Check In	Goal
**Medication:**	Took All	Take All
**Sleep Duration:**	8 hours	8.5 - 10.5 hours
**Bedtime:**	11:30 PM	9:00 PM - 10:30 PM
**Risetime:**	8:00 AM	6:30 AM - 8:00 AM
**Wellness Rating:**	Balanced (0)	-1 to +1

Weekly Summary

**Domains**	**Metric**		**06/17/19**	**06/10/19**	**06/03/19**	**05/27/19**	**05/20/19**	**05/13/19**	**05/06/19**	**04/29/19**	**04/22/19**	**04/15/19**	**04/08/19**	**04/01/19**	**03/25/19**
Weekly Check In	Survey(AMRS) >=6 possible mania		0	0	0	0	3	2	0	0	0	0	0	0	0
Mania	Early Warning Signs, 8 maximum		0	0	0	0	3	3	0	0	0	0	0	0	0
Weekly Check In	Survey(PHQ8) >=10 possible depression		1	0	1	6	0	3	4	6	3	3	1	4	6
Depression	Early Warning Signs, 8 maximum		0	0	0	6	0	1	0	4	1	1	0	2	3
Daily Check Ins	Days Reported, 7 maximum		7	7	7	7	7	7	7	7	7	7	7	7	4
Sleep	Duration Min, hours		2	5	4.5	4	5.5	5	6	5.5	6	8	8.5	6.5	6
	Duration Mean, hours		8	8.5	8.5	7.5	8.5	8.5	9	8.5	8.5	9.5	9.5	9	8.5
	Duration Max, hours		10	10.5	10	10.5	10.5	10	11	10	10	10	11	11	10
	In Range, %		43	71	71	57	71	71	43	57	57	71	86	57	75
Routine	In Bedtime Window, %		71	86	71	57	100	57	100	71	100	100	57	43	100
	In Risetime Window, %		71	71	14	29	43	71	71	71	57	71	57	71	100
Medications	Adherence, %		100	100	100	93	100	93	100	100	93	100	100	100	100
Wellness	Moderate Up, %	(+3, Episode)	0	0	0	0	0	0	0	0	0	0	0	0	0
	Mild Up, %	(+2, Prodromal)	0	0	0	0	14	0	0	0	0	0	0	0	0
	Well, %	(-1 to +1, Balanced)	100	100	86	57	86	100	71	57	100	100	100	100	100
	Mild Down, %	(-2, Prodromal)	0	0	14	43	0	0	29	43	0	0	0	0	0
	Moderate Down, %	(-3, Episode)	0	0	0	0	0	0	0	0	0	0	0	0	0

LiveWell Clinical Status Summary

Example User 3

Daily Summary for July 14, 2019

Clinical Status:

Recommendation:

	Daily Check In	Goal
**Medication:**		Take All
**Sleep Duration:**		6.5 - 8.5 hours
**Bedtime:**		8:30 PM - 10:00 PM
**Risetime:**		3:00 AM - 4:30 AM
**Wellness Rating:**		-1 to +1

Weekly Summary

Domains	Metric		07/08/19	07/01/19	06/24/19	06/17/19	06/10/19	06/03/19	05/27/19	05/20/19	05/13/19	05/06/19	04/29/19	04/22/19	04/15/19
Weekly Check In	Survey(AMRS) >=6 possible mania			3	4	3	3	1	5	2	6	4	3	5	5
Mania	Early Warning Signs, 8 maximum			1	1	2	1	2	3	3		3	1	4	3
Weekly Check In	Survey(PHQ8) >=10 possible depression			5	5	7	5	7	7	7	3	5	6	6	9
Depression	Early Warning Signs, 8 maximum			3	3	4	1	4	3	3			4	2	
Daily Check Ins	Days Reported, 7 maximum		3	6	6	7	7	7	7	7	7	7	6	5	4
Sleep	Duration Min, hours		6	6	5.5	6	6	6.5	6.5	6.5	6.5	7.5	6	5	5.5
	Duration Mean, hours		6.5	6.5	7	7	7.5	7.5	7	6.5	7	7.5	8	7	8
	Duration Max, hours		7.5	8	8	8	8	8.5	8	7.5	7.5	8	8.5	9	9
	In Range, %		33	83	67	86	86	100	100	100	100	100	83	60	75
Routine	In Bedtime Window, %		100	100	67	57	57	86	86	100	57	86	50	100	0
	In Risetime Window, %		67	83	67	43	14	43	71	100	71	71	33	40	0
Medications	Adherence, %		100	100	100	93	100	100	100	100	86	100	100	100	100
Wellness	Moderate Up, %	(+3, Episode)	0	0	0	0	0	0	0	0	0	0	0	0	0
	Mild Up, %	(+2, Prodromal)	0	0	0	0	0	0	0	0	57	0	0	0	25
	Well, %	(-1 to +1, Balanced)	100	100	100	100	100	100	100	100	43	100	100	100	50
	Mild Down, %	(-2, Prodromal)	0	0	0	0	0	0	0	0	0	0	0	0	25
	Moderate Down, %	(-3, Episode)	0	0	0	0	0	0	0	0	0	0	0	0	0
