# Supplementary material for: A Smartphone-Based Self-management Intervention for Individuals With Bipolar Disorder (LiveWell): Protocol Development for an Expert System to Provide Adaptive User Feedback
Source: JMIR Form Res. 2021 Dec 24;5(12):e32932. doi: 10.2196/32932 (PMC8742209; doi:10.2196/32932)
Supplement: Multimedia Appendix 5 [file formative_v5i12e32932_app5.pdf]

Multimedia Appendix 5. Psychiatrist Survey Summary

Under condition below, the LiveWell application	should instruct user to call you	should email you an urgent alert
**Symptoms**		
Not in a crisis but reports multiple symptoms of mania for	2-3 days	4 days*
Not in a crisis but reports multiple symptoms of depression for	3-4 days	#
Exhibits 1-2 early warning signs for	3-4 days	#
Physician Health Questionnaire 8 score was	15-20	#
Altman Self-Rating Mania Scale score was	10-16	6~
**Medication adherence**		
On average over 7 days, takes medications for	3 days*	3-5 days
On average over 4 days, takes medications for	1-2 days	0-2 days
**Sleep duration**		
Sleeps only 0-2 hours for	2 days*	3 days*
Sleeps only 2-4 hours for	2-3 days	2-4 days
On average over 7 days, sleep duration is LESS than usual by	3-4 hours	3-4 hours
On average over 3 days, sleep duration is LESS than usual by	3-4 hours	4 hours
On average over 7 days, sleep duration is MORE than usual by	4 hours*	4 hours
On average over 3 days, sleep duration is MORE than usual by	4 hours	#

*Only meets cumulative percent response criteria of ≥ 80%. ~Only meets mode percent response criteria of mode ≥ 10% > than pre and post mode response options. #Does not meet cumulative or mode percent response criteria. When range present, mode percent response sets lower limit and cumulative percent response sets upper limit.
