# Supplementary material for: A Smartphone-Based Self-management Intervention for Individuals With Bipolar Disorder (LiveWell): Protocol Development for an Expert System to Provide Adaptive User Feedback
Source: JMIR Form Res. 2021 Dec 24;5(12):e32932. doi: 10.2196/32932 (PMC8742209; doi:10.2196/32932)
Supplement: Multimedia Appendix 6 [file formative_v5i12e32932_app6.pdf]

Multimedia Appendix 6. Daily Review Decision Rules

CCN	Content Category*		Clinical Status	Daily Check In	Last 4 Daily Check Ins
1-2	Crisis		Any	|WR| = 4	
3-4	Episode	Continuing	Unwell	|WR| = 3	
5-7		Improving		|WR| < 3	
8-9	Worsening Symptoms		Not Unwell	|WR| = 3	
10-11	Recovering	Continuing	Recovering	|WR| = 2	
12-13	Prodromal		Prodromal		
14	Recovering	Improving	Recovering	|WR| < 2	
15	Prodromal		Prodromal		
16-17	Early Warning Signs		Well	|WR| = 2	
18	High Risk	Medication Adherence	Well	Medications ≠ All	#(Medications ≠ All) ≥ 3
19		Sleeping Too Little		Sleep Less-Severe	#(Sleep Less-Severe) ≥ 2
20		Sleeping Too Much		Sleep More-Severe	#(Sleep More-Severe) ≥ 3
21	Moderate Risk	Medication Adherence	Well	Medications ≠ All	
22		Sleeping Too Little		Sleep Less or Less-Severe	#(Sleep Less or Less-Severe) ≥ 2 & #(Sleep More or More-Severe) ≤ 1
23		Sleeping Too Much		Sleep More or More-Severe	#(Sleep More or More-Severe) ≥ 2 & #(Sleep Less or Less-Severe) ≤ 1
24		Sleeping Erratically		Sleep Less, Less-Severe, More, or More-Severe	#(Sleep Less, Less-Severe, More, More-Severe) ≥ 3
25		Irregular Routine		Missed Bedtime or Risetime Window	#(Missed Bedtime & Risetime Windows) ≥ 6
26	Low Risk		Well		

CCN = Content category number. *If criteria for row not met, move down to next row. |WR| = absolute value of wellness rating. #(X) = count of daily check ins during interval satisfying condition. Sleep Less-Severe: ≤ 4 hours of sleep. Sleep More-Severe: ≥ 12 hours of sleep or ≥ personalized goal upper limit plus 4 hours, whichever is less. Sleep Less: < personalized goal lower limit (eg 6 hours). Sleep More: > personalized goal upper limit (eg 8 hours). Bedtime Window: personalized 1.5 hour window for going to bed (eg 10:30 pm to MN). Risetime Window: personalized 1.5 hour window for getting up to start day (eg 7:00 to 8:30 am).
