# Supplementary material for: A Smartphone-Based Self-management Intervention for Individuals With Bipolar Disorder (LiveWell): Protocol Development for an Expert System to Provide Adaptive User Feedback
Source: JMIR Form Res. 2021 Dec 24;5(12):e32932. doi: 10.2196/32932 (PMC8742209; doi:10.2196/32932)
Supplement: Multimedia Appendix 7 [file formative_v5i12e32932_app7.pdf]

Multimedia Appendix 7 Clinical and Adherence Reachout Decision Rules

Clinical Reachout Category*		Clinical Status	Daily Check In		Last 7 Daily Check Ins	Last 4 Daily Check Ins
Crisis	Up	Any	WR = 4			
	Down		WR = -4			
Episode	Mania	To Unwell	WR = 3		#(WR ≥ 3) > #(WR ≤ -3)	
	Depression		WR = -3		#(WR ≤ -3) > #(WR ≥ 3)	
	Mood					
Prodromal	Mania	To Prodromal	WR ≥ 2		#(WR ≥ 2) > #(WR ≤ -2)	
	Depression		WR ≤ -2		#(WR ≤ -2) > #(WR ≥ 2)	
	Symptoms					
Worsening Symptoms	Mania	Not Unwell	WR = 3		#(WR ≥ 3) ≥ 4	
						#(WR ≥ 3) ≥ 3
	Depression		WR = -3		#(WR ≤ -3) ≥ 4	
	Mixed		|WR| = 3		#(WR ≤ -3) ≥ 2	#(WR ≥ 3) ≥ 2
High Risk	Medications	Well	|WR| ≤ 1	Medications ≠ All		#(Medications ≠ All) = 4
	Medications**					#(Medications ≠ All) = 3
	Sleep Less			Sleep Less-Severe		#(Sleep Less-Severe) ≥ 3
	Sleep Less**					#(Sleep Less-Severe) = 2
	Sleep More			Sleep More-Severe		#(Sleep More-Severe) = 4
	Sleep More**					#(Sleep More-Severe) = 3

Clinical Reachout Category		Weekly Check In
Episode	Mania	Prior ASRM < 6 & New ASRM ≥ 6
	Depression	Prior PHQ8 < 10 & New PHQ8 ≥ 10

Adherence Reachout Category	Daily Check In Last 7 Days	Weekly Check In Last 14 Days
Daily Check In	Missed ≥ 3	
Weekly Check In		Missed = 2

*If criteria for row not met, move down to next row for daily and weekly check in and adherence separately. **Pop-up alert for participant without email alert to study staff or provider. |WR| = absolute value of wellness rating. #(X) = count of daily check ins during interval satisfying condition. Sleep Less-Severe: ≤ 4 hours of sleep. Sleep More-Severe: ≥ 12 hours of sleep or ≥ personalized goal upper limit plus 4 hours, whichever is less. ASRM = Altman Self-Rating Mania Scale. PHQ8 = Patient Health Questionnaire 8
